# Improvement in small-bowel and colonic lesions observed by intestinal ultrasonography in Crohn's disease treated with risankizumab

**DOI:** 10.1097/MD.0000000000047853

**Published:** 2026-02-28

**Authors:** Maki Miyakawa, Masanao Nasuno, Masanori Nojima, Airi Konno, Kazuki Shimoyama, Takahito Hamada, Kohei Sugiyama, Hiroki Tanaka

**Affiliations:** a Sapporo IBD Clinic, Sapporo, Japan; b Center for Translational Research, Institute of Medical Science Hospital, University of Tokyo, Tokyo, Japan; c Department of Radiology, Sapporo IBD Clinic, Sapporo, Japan; d Department of Gastroenterology, Sapporo Central Hospital, Sapporo, Japan.

**Keywords:** biologic therapy, bowel wall thickness, Crohn's disease, intestinal ultrasonography, risankizumab, small-bowel lesions

## Abstract

This retrospective, single-center cohort study aimed to investigate improvements in small-bowel and colonic lesions, as assessed by intestinal ultrasonography (IUS), in patients with Crohn's disease (CD) treated with risankizumab. All patients with CD who received risankizumab between February 2023 and August 2024 in a clinic in Japan were included, but those with a prior intestinal resection or normal bowel wall thickness (BWT) on IUS were excluded. For each patient, the segment with the greatest BWT served as the representative lesion. Temporal changes in IUS parameters, including BWT, bowel wall stratification, and superb microvascular imaging, were evaluated at baseline and at weeks 4, 8, 12, 20, and 28. The IUS parameters were then compared between patients grouped according to lesion location (colon, small bowel, terminal ileum, and proximal to the terminal ileum). In total, 33 patients with CD were included. The representative lesion was located in the small bowel and colon in 19 and 14 patients, respectively. Following risankizumab treatment, both the BWT and the BWT reduction rate significantly reduced over time. At week 28, the IUS response rate was 39.4%, while the BWT normalization rate was 24.2%. Additionally, the proportions of patients with superb microvascular imaging grades 0 and ≤1 increased significantly over time, reaching 66.7% and 93.9%, respectively. In terms of lesion location, both the BWT and BWT reduction rate greatly reduced in the colon group compared with those in the small-bowel group (−21.4% vs −17.1%). They also significantly reduced from week 20 onward in the proximal-to-terminal-ileum group. Therefore, risankizumab significantly improved the BWT and vascularity in patients with CD, even in small-bowel lesions proximal to the terminal ileum.

## 1. Introduction

Crohn's disease (CD) is a chronic inflammatory disease of the gastrointestinal tract marked by periods of relapse and remission; if it is inadequately treated, it can result in serious complications, such as strictures, fistulas, and abscesses, and may cause long-term symptoms including chronic diarrhea, abdominal pain, and fever.^[1,2]^ Treatment strategies that are focused solely on symptom control do not alter CD’s natural course.^[3]^ The Selecting Therapeutic Targets in Inflammatory Bowel Disease-II statement emphasized that a treat-to-target approach is important in managing inflammatory bowel disease (IBD), with clinical remission, endoscopic healing, quality-of-life restoration, and disability absence as the most important long-term treatment goals for patients with CD.^[4]^ In the Effect of Tight Control Management on CD (CALM) study, early induction of deep remission significantly reduced the risk of disease progression over a median follow-up of 3 years, reinforcing mucosal healing as a valid therapeutic target.^[5]^

Risankizumab is a humanized monoclonal antibody targeting the interleukin-23 p19 subunit that has been developed to treat moderate-to-severe CD. Pivotal phase 3 trials such as ADVANCE and MOTIVATE have adopted clinical remission and endoscopic response, which both reflect mucosal healing, as coprimary endpoints at week 12.^[6]^ In the ADVANCE trial, risankizumab demonstrated endoscopic response and remission rates of 40% and 24%, respectively, at week 12; both rates were significantly higher than those in the placebo group. However, ileocolonoscopy, which largely assesses the colon and terminal ileum, has been primarily used for evaluating these endoscopic outcomes. Consequently, the impact of risankizumab on small-bowel lesions, especially those located proximal to the terminal ileum, remains insufficiently assessed.

Intestinal ultrasonography (IUS) is a noninvasive, low-cost, and convenient imaging tool that generates results quickly, enabling rapid medical decision-making.^[7,8]^ Various studies have assessed the value of cross-sectional imaging techniques using IUS to monitor transmural inflammation in CD.^[9–11]^ IUS has been recognized as a valid tool for CD diagnosis, relapse detection, and treatment response monitoring by the European Crohn’s and Colitis Organization, the European Society of Gastrointestinal and Abdominal Radiology, and the European Federation of Societies for Ultrasound in Medicine and Biology.^[12,13]^ In a recent multicenter study, therapeutic response was associated with statistically significant reductions in bowel wall thickness (BWT) or bowel wall stratification (BWS) and increased color Doppler ultrasound signals.^[9]^ Reports evaluating the effectiveness of biologic agents using IUS are currently limited. However, Voogd et al prospectively monitored IUS findings obtained from the initiation of antitumor necrosis factor therapy in patients with CD and found that BWT improved significantly among patients with an endoscopic response (>50% improvement in the simple endoscopic score for CD [SES-CD]) compared with that in nonresponders.^[14]^ Furthermore, a substudy of the Study of Treat-to-Target Versus Routine Care Maintenance Strategies in CD Patients Treated with Ustekinumab Trial (STARDUST), which was an international, multicenter, phase 3b, randomized controlled trial investigating patients with CD treated with ustekinumab, used IUS to evaluate the effect of ustekinumab on transmural bowel inflammation in adults with moderate-to-severe CD.^[15]^ This substudy showed that by week 48, 46.3% of the patients exhibited a progressive response on IUS, defined as a reduction of 25% or more in BWT. Several biologic agents have been evaluated using IUS, but no studies have evaluated risankizumab for intestinal lesions using IUS.

In this study, we present the Risankizumab Cohort Evaluated by IUS in CD, a retrospective cohort study conducted at a single IBD-specialized clinic in Japan. This study primarily aimed to investigate the therapeutic effects of risankizumab on small-bowel and colonic lesions in patients with CD, using IUS as the primary imaging tool.

## 2. Methods

### 2.1. Study patients

This retrospective, single-center cohort study included all patients with CD who received their first risankizumab dose between February 2023 and August 2024, with their lesions evaluated by IUS at baseline and weeks 4, 8, 12, 20, and 28 at our clinic. All patients underwent ileocolonoscopy before risankizumab initiation. CD diagnosis was based on the criteria determined by the Japanese Ministry of Health, Labor, and Welfare.^[16]^ We excluded patients with a history of intestinal resection or normal BWT on IUS. Included patients received a 600-mg induction dose intravenously at weeks 0, 4, and 8, followed by a 360-mg subcutaneous maintenance dose every 8 weeks using an autoinjector device.

### 2.2. Data collection

All data were obtained from the medical records at our institution. We accessed patient records and data from May 1, 2025, to July 1, 2025. Since the study involved collecting data from each patient’s medical records, the authors had access to information that could identify individual participants during the data collection period. Demographic data collected at risankizumab therapy initiation included sex, age, disease duration before risankizumab therapy, disease location (ileitis, ileocolitis, or colitis), body weight, disease behavior, active perianal disease presence, Harvey–Bradshaw Index (HBI) score,^[17]^ C-reactive protein (CRP), albumin, leucine-rich alpha-2 glycoprotein (LRG) levels, and previous use of biologic agents (e.g., infliximab, adalimumab, ustekinumab, and vedolizumab) or small-molecule agents (e.g., upadacitinib). We also documented concomitant medications at risankizumab initiation, including 5-aminosalicylicacid, prednisolone, budesonide, immunomodulators (azathioprine or 6-mercaptopurine), and elemental diet therapy of more than 300 kcal with Elental (EA Pharma, Tokyo, Japan). No patients received concomitant treatment with other biologic agents or small-molecule agents during risankizumab therapy. The presence of drainage from the fistula tract indicated active perianal disease. At ileocolonoscopy before risankizumab initiation, we recorded the SES-CD. Disease behavior was categorized according to the Montreal classification as follows: nonstricturing and nonpenetrating (B1), stricturing (B2), and penetrating (B3).^[18]^ As determined by each physician, indications for initiating risankizumab therapy were classified into “symptomatic activity” (clinical symptoms with HBI ≥ 5) or “endoscopic activity” (endoscopic activity in patients without symptomatic activity; HBI < 5). An HBI score below 5 also indicated symptomatic remission. All adverse events (AEs), including any malfunctions or issues related to the auto-injector device, during the study period were recorded.

### 2.3. IUS examination

All examinations were performed by 1 of 3 radiological technologists using Aplio a550 (Canon Medical Systems Corp., Tochigi, Japan) at baseline and at weeks 4, 8, 12, 20, and 28. The investigators had different levels of experience in IUS: 1 with more than 10 years, 1 with more than 2 years, and 1 with <2 years. Among them, 1 technologist was a certified medical sonographer accredited by the Japan Society of Ultrasonics in Medicine, while the other 2 technologists did not hold formal sonographer certification. Although formal interobserver reliability was not assessed, all examinations were conducted according to a predefined standardized protocol. Patients were examined regardless of their fasting status. During the IUS examination, the bowel was scanned from the ileum to all colonic segments.^[19,20]^ Small-bowel scanning began by locating the terminal ileum anterior to the psoas muscle and then following its course proximally as far as possible. The scan was initiated using a 5-MHz convex probe, which was subsequently switched to a 9.2-MHz linear probe for higher-resolution assessment. BWT was measured from the interface echo between the serosa and the muscularis propria to that between the lumen and the mucosa. Furthermore, superb microvascular imaging (SMI) was used for evaluating blood flow signals in the intestinal wall. The SMI parameter settings were as follows: color velocity, 1.2 cm/s; color frequency, 4 MHz.

### 2.4. IUS parameters

Each intestinal segment (small bowel, ascending colon, transverse colon, descending colon, and sigmoid colon), excluding the rectum, was imaged using IUS. The IUS findings were analyzed using the following IUS parameters: BWT, BWS, and SMI signals. Active BWT (BWT ≥ 3 mm for the small bowel or BWT ≥ 4 mm for the colon) indicated disease activity.^[12]^ The small bowel was divided into 2 segments: the terminal ileum (the region within 10 cm from the ileocecal valve) and the proximal to the terminal ileum (the proximal small bowel excluding the terminal ileum). BWS was categorized as preserved or lost. For SMI signals, we used a modified scoring system based on the Limberg score.^[21]^ This system was categorized into 4 grades (grade 0, no vascularization; grade 1, short stretches of vascularity appearing as spots; grade 2, longer stretches of vascularity; and grade 3, longer stretches of vascularity reaching the mesentery; Figure S1, Supplemental Digital Content, https://links.lww.com/MD/R469), as previously described in our earlier report.^[22]^ Active IUS findings were defined as active BWT, absent BWS, or SMI grade ≥1. The findings of BWT, BWS, and SMI signals for each intestinal segment were recorded. For each patient, the segment with the greatest BWT was used as the representative lesion. We also evaluated the IUS response (≥25% BWT reduction from the baseline or BWT normalization) based on the criteria used in the STARDUST trial.^[15]^

### 2.5. Assessment of the clinical outcome with risankizumab

We evaluated the median values of HBI scores, as well as the proportion of patients achieving symptomatic remission, from baseline to weeks 4, 8, 12, 20, and 28 in all patients and those with a baseline HBI score of 5 or more. The median CRP levels were also evaluated at the same time points. The median LRG levels were evaluated at baseline, week 12, and week 28. Among the patients with active draining perianal fistulas at baseline, the proportion achieving drainage resolution was evaluated at weeks 4, 8, 12, 20, and 28. The proportions of patients with active draining perianal fistulas in all patients at baseline and at weeks 4, 8, 12, 20, and 28 were also evaluated.

### 2.6. Assessment of improvement in small-bowel and colonic lesions with risankizumab

The primary endpoint was the IUS response rate at week 28. The secondary endpoints were changes in BWT, BWT reduction rate, IUS response rate, BWT normalization rate, and the proportions of patients with preserved BWS and SMI grades 0 and ≤1 from baseline to week 28. Additionally, the IUS parameters regarded as the secondary endpoints were compared between patients with and without a history of biologic therapy. For subgroup analysis, patients were divided into 2 groups according to the location of the representative lesion at baseline: the small-bowel group and the colon group. The abovementioned IUS parameters (secondary endpoints) were also compared between these 2 groups. Similarly, within the small-bowel group, patients were further categorized into the terminal-ileum group and the proximal-to-terminal-ileum group to evaluate differences between lesion locations; their BWT and BWT reduction rate were also compared.

### 2.7. Statistical analysis

We present categorical variables as frequencies and percentages, and continuous variables as medians and interquartile ranges (IQRs). We used the Clopper–Pearson exact method to calculate the 95% confidence intervals for a fraction of patients. Furthermore, the Wilcoxon signed-rank test was employed to compare continuous variables between baseline and weeks 4, 8, 12, 20, and 28. To evaluate changes in the proportions of categorical variables between such time points, we used the McNemar test. The categorical variables were also compared using the Fisher exact test. A *P*-value below .05 was considered statistically significant. Statistical multiplicity resulting from multiple testing across different time points was adjusted using Bonferroni correction. No adjustment was made for multiple outcomes because many of the outcomes were correlated, and such correction would have been overly conservative. Missing values were imputed using the last-observation-carried-forward (LOCF) method. For patients undergoing risankizumab discontinuation or surgery during the study period, missing data were imputed using the LOCF method according to the last risankizumab administration before discontinuation or surgery. All statistical data were analyzed using the Statistical Package for the Social Sciences software, version 31.0 (SPSS Inc., Chicago, IL) and EZR (Saitama Medical Center, Jichi Medical University, Saitama, Japan).

### 2.8. Ethical considerations

The ethics committee of Sapporo IBD Clinic approved this study (August 20, 2024; approval number: 2025-12). The study was conducted following the tenets of the Declaration of Helsinki. Given the study’s retrospective nature, written informed consent was waived. Information regarding the study was available to the patients on the institution’s website, and the patients had the right to cease registration of their data at any time.

## 3. Results

### 3.1. Patient characteristics

We identified 50 patients with CD who received risankizumab within the study period. After excluding 11 patients with a history of intestinal resection and 6 with normal BWT on IUS, 33 were finally included in the analysis. Table 1 summarizes study participants’ baseline characteristics at first risankizumab administration. Among the 33 patients, only 8 were female. The median age was 26.0 years, and the median disease duration was 2.0 years. At baseline, the median HBI score was 5, and the median CRP and LRG levels were 0.67 mg/dL and 19.9 μg/mL, respectively. Active perianal disease was found in 13 patients. The risankizumab therapy was initiated because of “symptomatic activity” in 18 patients and “endoscopic activity” in 15. The median SES-CD score was 15.0, and the median interval from ileocolonoscopy to baseline was 21 (IQR: 9.0–45.5) days. The representative lesion with the greatest BWT was located in the small bowel in 19 patients and the colon in 14. Of the small-bowel lesion cases, 7 involved the terminal ileum and 12 involved segments proximal to the terminal ileum. The median BWT at baseline was 5.2 mm. Additionally, BWS was lost in 24 patients and preserved in only 9 patients. Meanwhile, 3, 11, 14, and 5 patients obtained SMI grades of 0, 1, 2, and 3, respectively.

**Table 1:** Baseline characteristics of the 33 patients with Crohn's disease.

Variable	n = 33
Female/male, n (%)	8 (24.2%)/25 (75.8%)
Median age, yr (IQR)	26.0 (21.5–36.0)
Median disease duration, yr (IQR)	2.0 (0–6.5)
Location of disease, n (%)	
Ileitis	2 (6.1%)
Ileocolitis	29 (87.9%)
Colitis	2 (6.1%)
Disease behavior, n (%)	
Nonstricturing, nonpenetrating (B1)	24 (72.7%)
Stricturing (B2)	9 (27.3%)
Penetrating (B3)	0 (0%)
Perianal disease, n (%)	28 (84.8%)
Active	13 (39.4%)
Inactive	15 (45.5%)
Harvey–Bradshaw Index, median (IQR)	5 (3–7.5)
C-reactive protein (mg/dL), median (IQR)	0.67 (0.16–2.21)
Albumin (g/dL), median (IQR)	3.9 (3.7–4.1)
Leucine-rich alpha-2 glycoprotein (μg/mL), median (IQR)	19.9 (15.9–31.3)
Indications for initiating risankizumab	
Symptomatic activity	18 (54.5%)
Endoscopic activity	15 (45.5%)
Simple endoscopic score for Crohn’s disease	15.0 (9.0–22.0)
Median interval from ileocolonoscopy to baseline, d (IQR)	21 (9.0–45.5)
Most affected segment, n (%)	
Small bowel	19 (57.6%)
Terminal ileum	7 (21.2%)
Proximal to the terminal ileum	12 (36.4%)
Colon	14 (42.4%)
Bowel wall thickness (mm), median (IQR)	5.2 (4.4–5.9)
Small bowel (n = 19)	5.2 (4.3–6.4)
Colon (n = 14)	5.1 (4.4–5.9)
Bowel wall stratification, n (%)	
Preserved	9 (27.3%)
Lost	24 (72.7%)
Superb microvascular imaging grade, n (%)	
Grade 0	3 (9.1%)
Grade 1	11 (33.3%)
Grade 2	14 (42.4%)
Grade 3	5 (15.2%)
Concomitant medications, n (%)	
Oral 5-aminosalicylic acid	27 (81.8%)
Prednisolone	15 (45.5%)
Budesonide	7 (21.2%)
Immunomodulators	10 (30.3%)
Azathioprine	9 (27.3%)
6-mercaptopurine	2 (6.1%)
Elemental diet therapy	13 (39.4%)
Previous medications, n (%)	
Infliximab	7 (21.2%)
Adalimumab	12 (36.4%)
Ustekinumab	7 (21.2%)
Vedolizumab	4 (12.1%)
Biologics	17 (51.5%)
Upadacitinib	0 (0%)

IQR = interquartile range.

### 3.2. Clinical outcomes with risankizumab

Among the 18 patients with a baseline HBI score of 5 or more, the median HBI score significantly decreased from 7.0 at baseline to 4.0, 3.5, 3.5, 3.0, and 3.5 at weeks 4, 8, 12, 20, and 28, respectively. The proportion of patients achieving symptomatic remission increased to 55.6%, 55.6%, 66.7%, 72.2%, and 72.2% at weeks 4, 8, 12, 20, and 28, respectively. Among all 33 patients, the median CRP levels significantly decreased from 0.67 mg/dL at baseline to 0.27, 0.27, 0.29, 0.22, and 0.19 mg/dL at weeks 4, 8, 12, 20, and 28, respectively. The median LRG levels significantly decreased from 19.9 μg/mL at baseline to 15.5 and 14.4 μg/mL at weeks 12 and 28, respectively. Among the 13 patients who had active draining perianal fistulas at baseline, drainage was resolved in 7.7%, 23.1%, 23.1%, 30.8%, and 38.5% of these patients at weeks 4, 8, 12, 20, and 28, respectively. In all 33 patients, the proportions of those with active draining perianal fistulas at baseline and at weeks 4, 8, 12, 20, and 28 were 39.4%, 36.4%, 30.3%, 30.3%, 27.3%, and 24.2%, respectively, with no statistically significant difference. Figures S2 to S4, Supplemental Digital Content, https://links.lww.com/MD/R469, depict the changes in HBI and symptomatic remission rate, CRP and LRG values, and perianal fistula status over time, respectively. LOCF imputation was applied in 4 patients (12.1%). Imputation was performed from week 8 onward in 1 patient with primary nonresponse, from week 12 onward in 1 patient who underwent surgery, and at week 28 in 2 patients (1 with primary nonresponse and 1 who discontinued follow-up). The number of patients with imputed data at each time point was 0 (0%) at week 4, 1 (3.0%) at week 8, 2 (6.1%) at weeks 12 and 20, and 4 (12.1%) at week 28.

### 3.3. BWT changes, IUS response, and BWT normalization with risankizumab

**Figure 1.**
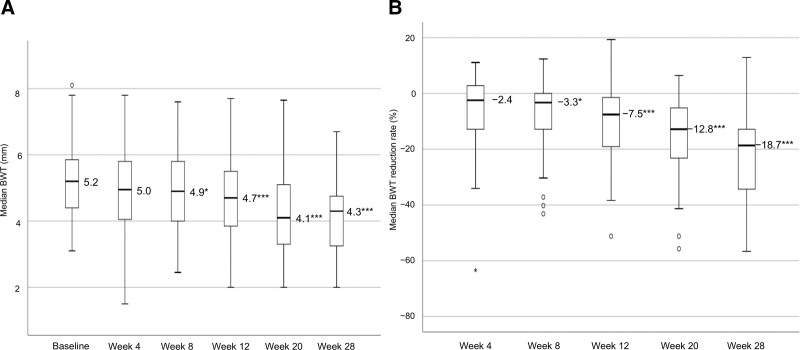
Median BWT and median BWT reduction rate. (A) Changes in the median BWT. (B) Changes in the median BWT reduction rate. Missing values were imputed using the LOCF method. *P*-values were determined using the Wilcoxon signed-rank test (change from baseline, *P < .05, ***P < .001). Statistical multiplicity was adjusted using Bonferroni correction. BWT = bowel wall thickness, LOCF = last-observation-carried-forward.

**Figure 2.**
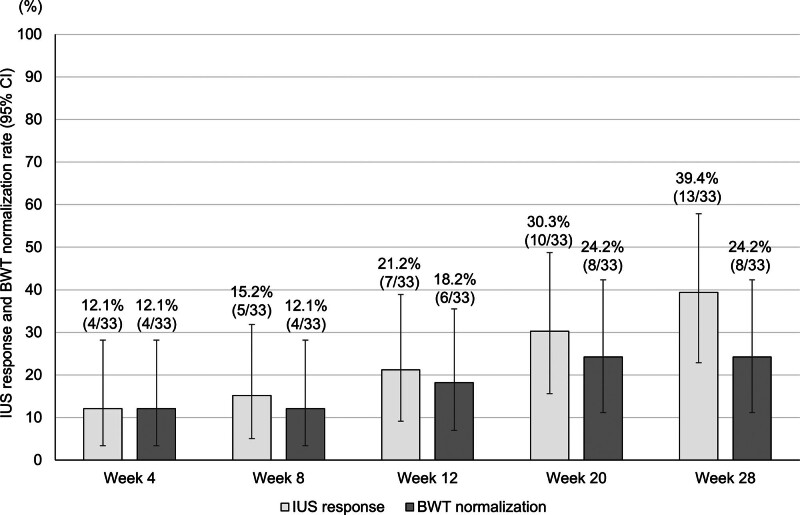
IUS response rate and BWT normalization rate. Changes in the IUS response rate and the BWT normalization rate. Missing values were imputed using the LOCF method. BWT = bowel wall thickness, CI = confidence interval, IUS = intestinal ultrasonography, LOCF = last-observation-carried-forward.

Figure 1 shows the changes in the median BWT from baseline to week 28 and the BWT reduction rate from weeks 4 to 28, following risankizumab initiation. The median BWT values at baseline and weeks 4, 8, 12, 20, and 28 were 5.2, 5.0, 4.9, 4.7, 4.1, and 4.3 mm, respectively, and the BWT reduction rates at weeks 4, 8, 12, 20, and 28 were −2.4%, −3.3%, −7.5%, −12.8%, and −18.7%, respectively. As observed, both the BWT and the BWT reduction rate decreased over time, with significant reductions observed from week 8 onward. Moreover, the IUS response rates at weeks 4, 8, 12, 20, and 28 were 12.1%, 15.2%, 21.2%, 30.3%, and 39.4%, respectively, and the corresponding BWT normalization rates were 12.1%, 12.1%, 18.2%, 24.2%, and 24.2%, respectively, which showed a gradual increase over time (Fig. 2).

### 3.4. BWS and vascularity changes with risankizumab

**Figure 3.**
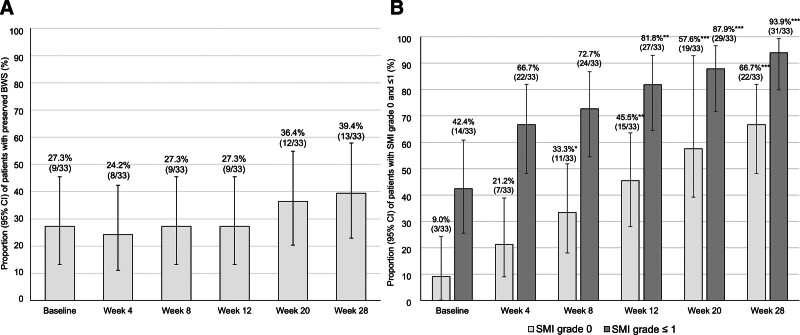
Proportion of preserved BWS and SMI grades 0 and ≤1. (A) Changes in the proportion of patients with preserved BWS. (B) Changes in the proportion of patients with SMI grades 0 and ≤1. Missing values were imputed using the LOCF method. *P*-values were determined using the McNemar test (change from baseline, *P < .05, **P < .01, ***P < .001). Statistical multiplicity was adjusted using Bonferroni correction. BWS = bowel wall stratification, CI = confidence interval, LOCF = last-observation-carried-forward, SMI = superb microvascular imaging.

BWS was preserved in 27.3%, 24.2%, 27.3%, 27.3%, 36.4%, and 39.4% of patients at baseline and at weeks 4, 8, 12, 20, and 28, respectively. Although it slightly increased after week 20, this change was not statistically significant (Fig. 3A). At the same time points, SMI grade 0 was observed in 9.0%, 21.2%, 33.3%, 45.5%, 57.6%, and 66.7% and grade ≤1 in 42.4%, 66.7%, 72.7%, 81.8%, 87.9%, and 93.9% of patients, respectively. The proportion of these SMI grades increased over time, with a significant increase observed from week 8 onward for grade 0 and from week 12 onward for grade ≤1 (Fig. 3B).

### 3.5. IUS findings stratified by biologic therapy history

**Figure 4.**
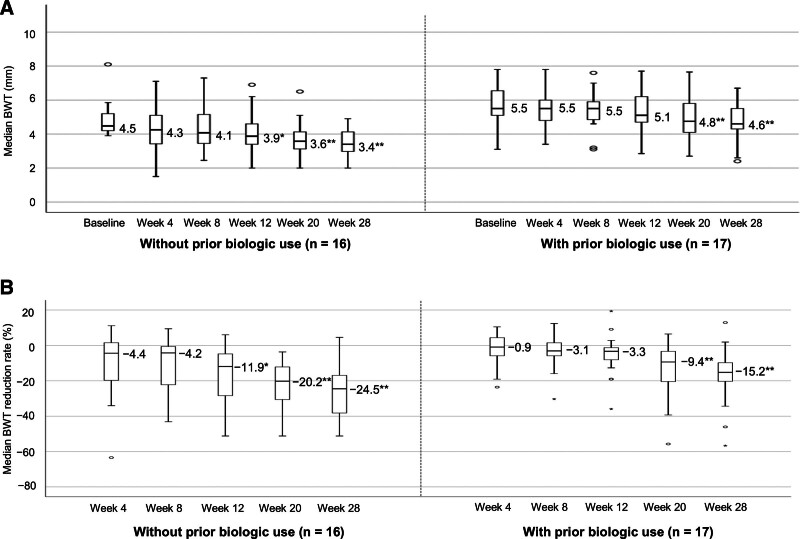
Median BWT and median BWT reduction rate stratified by biologic therapy history. (A) Changes in median BWT stratified by prior biologic use. (B) Changes in the median BWT reduction rate stratified by prior biologic use. Missing values were imputed using the LOCF method. *P*-values were determined using the Wilcoxon signed-rank test (change from baseline, *P < .05, **P < .01). Statistical multiplicity was adjusted using Bonferroni correction. BWT = bowel wall thickness, LOCF = last-observation-carried-forward.

**Figure 5.**
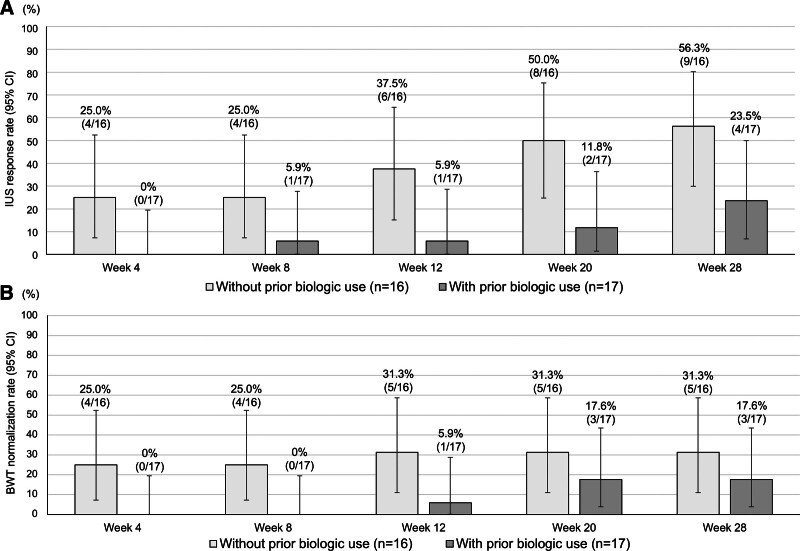
IUS response rate and BWT normalization rate stratified by biologic therapy history. (A) Changes in the IUS response rate stratified by prior biologic use. (B) Changes in the BWT normalization rate stratified by prior biologic use. Missing values were imputed using the LOCF method. BWT = bowel wall thickness, CI = confidence interval, IUS = intestinal ultrasonography, LOCF = last-observation-carried-forward.

Table S1, Supplemental Digital Content, https://links.lww.com/MD/R469, lists the baseline characteristics of patients with and without a biologic therapy history. Patients with prior biologic use (n = 17) had a significantly longer disease duration, greater BWT, and more frequent use of immunomodulators than those without prior biologic use (n = 16). Notably, in patients without a biologic therapy history, both BWT and the BWT reduction rate significantly improved as early as week 12, whereas in those with prior biologic use, these parameters significantly improved only from week 20 onward (Fig. 4). At week 28, the IUS response and BWT normalization rates increased among patients without prior biologic therapy (56.3% and 31.3%) compared with those among patients with biologic therapy history (23.5% and 17.6%; Fig. 5). The proportion of patients with preserved BWS increased slightly from 31.3% at baseline to 50.0% at week 28 in those without prior biologic use; conversely, the proportion slightly changed from 23.5% at baseline to 29.4% at week 28 in those with prior biologic use (Figure S5A, Supplemental Digital Content, https://links.lww.com/MD/R469). At week 28, the proportion of patients with SMI grades 0 and ≤1 increased compared with the baseline, rising from 17.6% and 58.8% to 58.8% and 94.1% in patients with prior biologic use and from 0% and 25.0% to 75.0% and 93.8% in those without prior biologic use, respectively (Figure S5B, Supplemental Digital Content, https://links.lww.com/MD/R469). Statistical analysis was not performed for SMI grade 0 in patients without prior biologic use because of a baseline value of 0. For SMI grades ≤1 and 0 in patients without and with prior biologic use, respectively, significant differences were noted between baseline and week 28.

### 3.6. Small-bowel and colonic lesion changes with risankizumab

**Figure 6.**
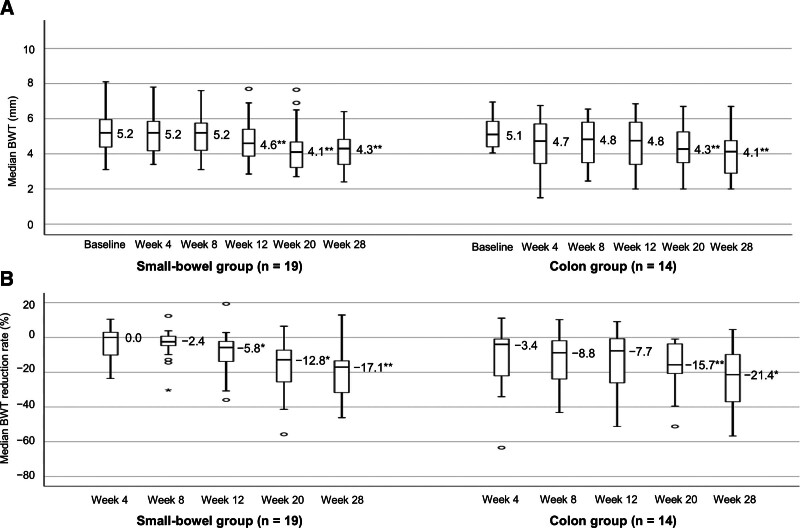
Median BWT and median BWT reduction rate stratified by the location of the representative lesion. (A) Changes in the median BWT stratified by the location of the representative lesion at baseline. (B) Changes in the median BWT reduction rate stratified by the location of the representative lesion at baseline. Missing values were imputed using the LOCF method. *P*-values were determined using the Wilcoxon signed-rank test (change from baseline, *P < .05, **P < .01). Statistical multiplicity was adjusted using Bonferroni correction. BWT = bowel wall thickness, LOCF = last-observation-carried-forward.

**Figure 7.**
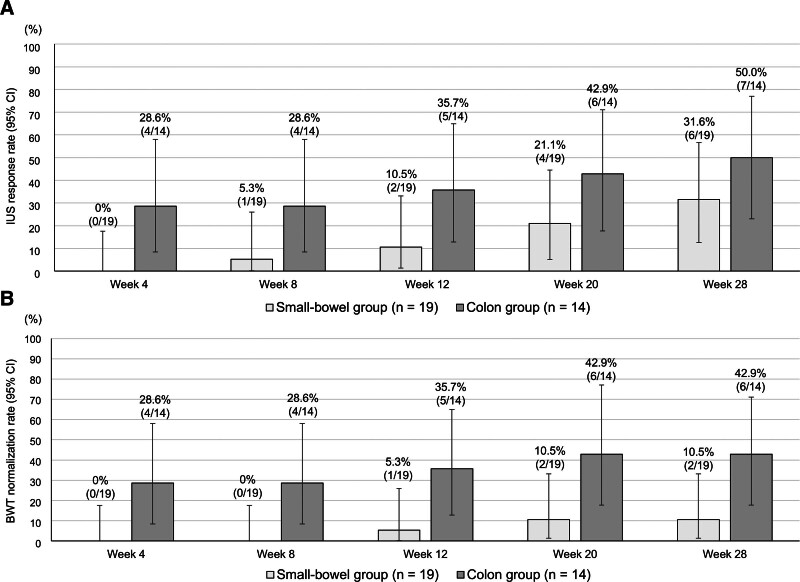
IUS response rate and BWT normalization rate stratified by the location of the representative lesion. (A) Changes in the IUS response rate stratified by the location of the representative lesion at baseline. (B) Changes in the BWT normalization rate stratified by the location of the representative lesion at baseline. Missing values were imputed using the LOCF method. BWT = bowel wall thickness, CI = confidence interval, IUS = intestinal ultrasonography, LOCF = last-observation-carried-forward.

Of the representative lesions, 19 were located in the small bowel and 14 in the colon. Accordingly, patients were classified into the small-bowel group (n = 19) and the colon group (n = 14). Table S2, Supplemental Digital Content, https://links.lww.com/MD/R469, lists the baseline characteristics of these 2 groups. Stricturing (B2) and BWS loss were significantly more frequent in the small-bowel group than those in the colon group. At baseline, the median BWT was 5.2 mm in the small-bowel group and 5.1 mm in the colon group. Both the BWT and the BWT reduction rate significantly improved from week 20 onward in the colon group and from week 12 onward in the small-bowel group (Fig. 6). At week 28, the BWT values were similar between the groups (small-bowel group, 4.3 mm; colon group, 4.1 mm), but the BWT reduction rate was greater in the colon group (−21.4% vs −17.1%). At the same week, the IUS response increased in the colon group (50.0%) compared with that in the small-bowel group (31.6%), with no statistically significant difference (Fig. 7A). In addition, the BWT normalization rate was numerically higher in the colon group (42.9%) than that in the small-bowel group (10.5%; Fig. 7B). At baseline, the colon group (50%) had a higher proportion of patients with preserved BWS than the small-bowel group (10.5%). By week 28, these proportions increased slightly to 57.1% and 26.3% in the colon and small-bowel groups, respectively, with no significant difference between baseline and week 28 in both groups (Figure S6A, Supplemental Digital Content, https://links.lww.com/MD/R469). Moreover, the proportions of patients with SMI grades 0 and ≤1 at week 28 increased compared with baseline, from 21.4% and 57.1% to 85.7% and 92.9% in the colon group and from 0% and 31.6% to 52.6% and 94.7% in the small-bowel group, respectively (Figure S6B, Supplemental Digital Content, https://links.lww.com/MD/R469). SMI grade 0 in the small-bowel group was not statistically analyzed because the baseline value was zero. Significant differences between baseline and week 28 were observed for SMI grade 0 in the colon group and SMI grade ≤1 in the small-bowel group, but not for SMI grade ≤1 in the colon group.

### 3.7. Terminal-ileum group versus proximal-to-terminal-ileum group

**Figure 8.**
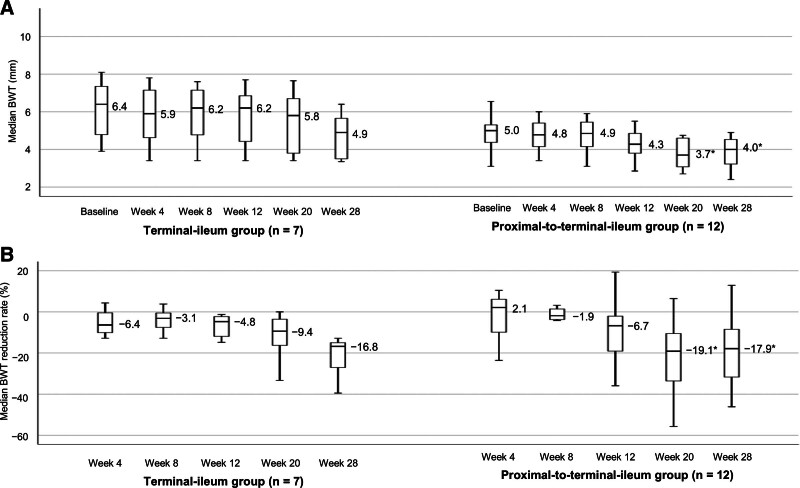
Median BWT and median BWT reduction rate stratified by the small-bowel subgroups. (A) Changes in the median BWT stratified by the small-bowel subgroups. (B) Changes in the median BWT reduction rate stratified by the small-bowel subgroups. Missing values were imputed using the LOCF method. *P*-values were determined using the Wilcoxon signed-rank test (change from baseline, *P < .05). Statistical multiplicity was adjusted using Bonferroni correction. BWT = bowel wall thickness, LOCF = last-observation-carried-forward.

In the terminal-ileum group, the median BWT values decreased from 6.4 mm at baseline to 4.9 mm at week 28, whereas in the proximal-to-terminal-ileum group, it decreased from 5.0 mm at baseline to 4.0 mm at week 28 (Fig. 8A). The BWT reduction rates in the terminal-ileum group were −6.4%, −3.1%, −4.8%, −9.4%, and −16.8% at weeks 4, 8, 12, 20, and 28, respectively, whereas those in the proximal-to-terminal-ileum group were 2.1%, −1.9%, −6.7%, −19.1%, and −17.9%, at the same time points (Fig. 8B). In the proximal-to-terminal-ileum group, BWT and the BWT reduction rate significantly decreased from week 20 onward.

### 3.8. AEs and autoinjector malfunctions

Table S3, Supplemental Digital Content, https://links.lww.com/MD/R469, enumerates all AEs occurring during the study period. The overall incidence of AEs was 21.2% (7/33). Two (6.0%) patients developed bowel obstruction. Risankizumab treatment was discontinued in 2 patients because of CD exacerbation. None of the participants developed serious infections, herpes zoster, or injection site reactions. Additionally, a total of 90 subcutaneous risankizumab injections were administered using the autoinjector, with only 1 malfunction reported (1.1%).

## 4. Discussion

To our knowledge, this study is the first to evaluate risankizumab effectiveness for intestinal lesions using IUS, providing valuable insights, especially for small-bowel lesions proximal to the terminal ileum. Although BWT and BWT reduction rate significantly improved from week 8 onward in our study, the degree of improvement was lower than that observed in the STARDUST substudy of patients with CD treated with ustekinumab.^[15]^ In the STARDUST substudy, early BWT reduction rates at weeks 4 and 8 were −9.55% and −11.89%, and the corresponding IUS response rates were 25.5% and 26.6%. However, this difference may be partly explained by methodological differences, as the STARDUST substudy used an as-observed analysis, whereas our study employed an LOCF approach. Notably, supplemental analysis of the STARDUST substudy using the nonresponder imputation (NRI) method reported the IUS response rates of 19.7%, 23.9%, 33.8%, and 35.2% at weeks 4, 8, 16, and 48, respectively, which were closer to those observed in our study.

The difference in IUS response rates between our study and the STARDUST substudy was particularly evident in patients with small-bowel lesions. The IUS response rates for the terminal ileum in the STARDUST substudy were 23.9%, 19.6%, 30.4%, and 32.6% at weeks 4, 8, 16, and 48, respectively, whereas those for the small bowel in our study were 0% at week 4 and 5.3% at week 8. By week 28, the IUS response in our small-bowel group increased to 31.6%, which was very similar to the terminal-ileum IUS response rates at weeks 16 (30.4%) and 48 (32.6%) in the STARDUST substudy. While the STARDUST substudy evaluated only the terminal ileum, our study assessed the entire small bowel. Notably, among the 19 patients with small-bowel lesions evaluated in our study, 12 (63%) had lesions proximal to the terminal ileum.

Moreover, our study showed that stricturing (B2) and BWS loss were more common in the small-bowel group than those in the colon group, indicating that the small-bowel group had more patients with advanced disease. In fact, compared with the small-bowel group, the colon group showed greater BWT reduction rates, had more patients with preserved BWS, had more patients who achieved SMI grade 0, and exhibited higher IUS response rates. Additionally, the colon group had a numerically higher rate of BWT normalization than the small-bowel group (42.9% vs 10.5%). Given the differences in patient backgrounds in this study, we cannot conclude whether colonic lesions are more likely to improve than small-bowel lesions, as assessed by IUS; however, some studies using balloon-assisted enteroscopy have reported that mucosal healing in small-bowel lesions is less likely than that in colonic lesions.^[23,24]^

To investigate the impact of lesions proximal to the terminal ileum, we separately evaluated lesions in the terminal ileum and those proximal to it. At baseline, the terminal-ileum group had a higher median BWT than the proximal-to-terminal-ileum group, but the value was similar to that of patients with colonic lesions. Nevertheless, the proximal-to-terminal-ileum group had the lowest BWT reduction rate up to week 8, particularly at weeks 4 and 8, compared with the terminal-ileum group and the colon group. This markedly poorer BWT reduction rate in the proximal-to-terminal-ileum group may have contributed to the lack of early improvement observed in our study. Interestingly, the BWT and BWT reduction rate significantly increased in the proximal-to-terminal-ileum group from week 20 onward. At week 28, the degree of BWT reduction in the proximal-to-terminal-ileum group was comparable with that observed in the terminal-ileum group. Therefore, while lesions proximal to the terminal ileum may exhibit poor early improvement, they can potentially improve over time. However, given that this subgroup analysis included very few patients, larger studies will be needed to clarify this issue.

In this study, of note, risankizumab led to rapid and substantial improvements in clinical outcomes such as CRP, LRG, active draining perianal fistulas, and HBI. Both CRP and LRG levels significantly decreased during follow-up. Interestingly, approximately 40% of patients with active draining perianal fistulas at baseline achieved drainage resolution during follow-up, a finding first demonstrated in this study to our knowledge. Among patients with an HBI score of 5 or more, the HBI score decreased significantly as early as week 4, with approximately 55% of patients achieving symptomatic remission. These HBI and symptomatic remission rate improvements are similar to those reported by Zinger et al in a single-center, real-world cohort of 80 patients wherein 61% achieved clinical remission at week 4 and 70% at week 12.^[25]^ Although real-world data on risankizumab use in patients with CD remain limited, Fumery et al reported in their multicenter retrospective cohort study that clinical remission was achieved in 45% of 152 patients at week 12 and 47% at week 26.^[26]^ Johnson et al also found that in their retrospective review of a multicenter consortium, 49.7% (98/197) of patients achieved clinical remission at week 12 and 37.9% (97/256) at 6 months.^[27]^ Moreover, Alsoud et al reported that in a Belgian multicenter cohort, 18.2% (10/55) of patients achieved clinical remission at week 24.^[28]^ Despite having a small sample size, our study obtained results that are not markedly inferior to those of previous reports, suggesting that risankizumab’s effectiveness was adequately demonstrated in our cohort.

However, the presence or absence of biologic therapy history influenced the efficacy of risankizumab. In ADVANCE, the subpopulation without previous biofailure showed higher efficacy than those with previous biofailure, including outcomes such as clinical remission and endoscopic remission.^[6]^ In this study, both BWT and the BWT reduction rate significantly improved in patients with and without a biologic therapy history. Notably, in patients with prior biologic use, both parameters significantly improved only from week 20 onward, whereas in those without prior biologic use, significant improvement was seen as early as week 12. Additionally, at week 28, all evaluated parameters were higher in patients without prior biologic use than in those with prior biologic use. Currently, reports on changes in IUS findings with biologic therapy stratified by prior biologic use are still few. In the STARDUST substudy, the mean percent change in BWT from baseline significantly decreased starting from week 4 in patients without prior biologic use and only from week 8 in those with prior exposure to a single biologic therapy.^[15]^ These BWT trends are similar to those in our study. Similarly, the IUS response rate at week 48 in the STARDUST substudy was 59.1% in biologic-naïve patients and 37.5% in those with prior exposure to 1 biologic therapy. These results are also similar to our study. However, patients with prior biologic use often have more challenging backgrounds. Similarly, patients with prior biologic use in our study had a significantly longer disease duration, greater BWT, and more frequent use of immunomodulators than those without prior biologic use. Therefore, these background differences should be considered when interpreting the greater improvement in IUS findings observed in biologic-naïve patients in this study.

Moreover, the proportion of patients with preserved BWS in our study increased from 27.3% at baseline to 39.4% at week 28, which is less favorable than the result obtained by the STARDUST substudy, where the proportion increased from 36.8% at baseline to 54.5% at week 16 and 64.2% at week 48.^[15]^ Notably, BWS improvement was greater in biologic-naïve patients, increasing from 31.3% at baseline to 50.0% at week 28, whereas in patients with prior biologic use, the proportion only slightly changed, rising from 23.5% at baseline to 29.4% at week 28. Nevertheless, the overall improvement in BWS in our study remained less favorable than that in the STARDUST substudy, and the reason behind it remains unclear. However, factors such as the frequency of the probe used and the physical condition of patients may affect BWS.^[20]^ Moreover, Novak et al revealed that for BWS, the interrater reliability of IUS parameters assessed by 12 IUS experts was low to moderate in patients with CD.^[29]^ Therefore, the BWS assessment may be better used when combined with other parameters.

In assessing vascularity, both our study and the STARDUST substudy showed similarly high improvement, regardless of whether patients had prior biologic use or not.^[15]^ In the STARDUST substudy, the proportion of patients without vascular signals (corresponding to color Doppler signal 0 or 1) increased from 37.3% at baseline to 61.3% and 72.5% at weeks 16 and 48, respectively. Although we used our own scoring system based on SMI, the proportion of our patients with SMI grade 0, which means no vascular signals, increased significantly from 9.0% at baseline to 66.7% at week 28, similar to the STARDUST substudy results. Notably, in our study, the improvement in SMI grade 0 was particularly noted among patients without prior biologic use, with 75.0% of them showing no vascular signals at week 28. Vascularity normalization tended to be higher than BWT normalization or preserved BWS in all patients, regardless of prior biologic use or the location of the lesion (small bowel vs colon). These findings suggest that vascularity normalization is the earliest indicator to be achieved as a treatment target for lesion improvement during risankizumab therapy in patients with CD within a treat-to-target strategy.

The strength of our study is that it is the first to evaluate risankizumab-treated patients with CD longitudinally by IUS, specifically assessing small-bowel lesions proximal to the terminal ileum. However, the study also has several limitations. First, given that this was a single-center cohort study, no specific IUS experience requirements were set for the 3 investigators, and the IUS findings were not independently assessed by multiple investigators. Second, IUS accuracy has inherent limitations, particularly in assessing small-bowel lesions. The insight into whether all small-bowel lesions were detected without omission and whether the exact same segments were consistently evaluated at each time point is uncertain. However, this concern may be minimal in our study, considering that no new lesions were detected during the observation period and the entire gastrointestinal tract underwent repeated, comprehensive assessments for each patient. Finally, we did not conduct endoscopic evaluation; thus, we could not directly compare IUS findings with the endoscopic measures of disease activity or mucosal healing. Nevertheless, IUS is an established tool for CD lesion assessment; therefore, the lack of endoscopic evaluation as a limitation may not have significantly affected the overall interpretation of our results. However, the Risankizumab Cohort Evaluated by IUS in CD study findings can provide useful information to clinicians in monitoring patients with CD treated with risankizumab using IUS.

In conclusion, this retrospective cohort study conducted at a single IBD-specialized clinic in Japan demonstrated that, as assessed by IUS, risankizumab treatment significantly improved BWT and vascularity in patients with CD, including those with small-bowel lesions proximal to the terminal ileum. These findings provide real-world evidence that risankizumab can improve both small-bowel and colonic lesions, as well as those proximal to the terminal ileum, in patients with CD as evaluated by IUS. Further prospective studies that include larger and more diverse populations are warranted to validate these results.

## Acknowledgments

The authors would like to thank all the patients enrolled in the RICE study. The authors would also like to thank Enago (www.enago.jp) for the English language review.

## Author contributions

**Conceptualization:** Maki Miyakawa.

**Data curation:** Maki Miyakawa, Masanao Nasuno, Kohei Sugiyama, Hiroki Tanaka.

**Formal analysis:** Maki Miyakawa, Masanao Nasuno, Masanori Nojima, Hiroki Tanaka.

**Investigation:** Maki Miyakawa, Masanao Nasuno, Airi Konno, Kazuki Shimoyama, Takahito Hamada.

**Supervision:** Hiroki Tanaka.

**Writing – original draft:** Maki Miyakawa, Masanao Nasuno, Hiroki Tanaka.

**Writing – review & editing:** Maki Miyakawa, Masanao Nasuno, Kohei Sugiyama, Hiroki Tanaka.

## Supplementary Material


