# Supplementary material for: Improvement in small-bowel and colonic lesions observed by intestinal ultrasonography in Crohn's disease treated with risankizumab
Source: Medicine (Baltimore). 2026 Feb 28;105(9):e47853. doi: 10.1097/MD.0000000000047853 (PMC12956175; doi:10.1097/MD.0000000000047853)
Supplement: Supplementary file 1 [file medi-105-e47853-s001.docx]

**Supplemental Digital Contents**

Figure S1.

Examples of SMI signals categorized into four grades. (a) Grade 0: no vascularization, (b) grade 1: short stretches of vascularity appearing as spots, (c) grade 2: longer stretches of vascularity, (d) grade 3: longer stretches of vascularity reaching the mesentery. SMI = superb microvascular imaging.


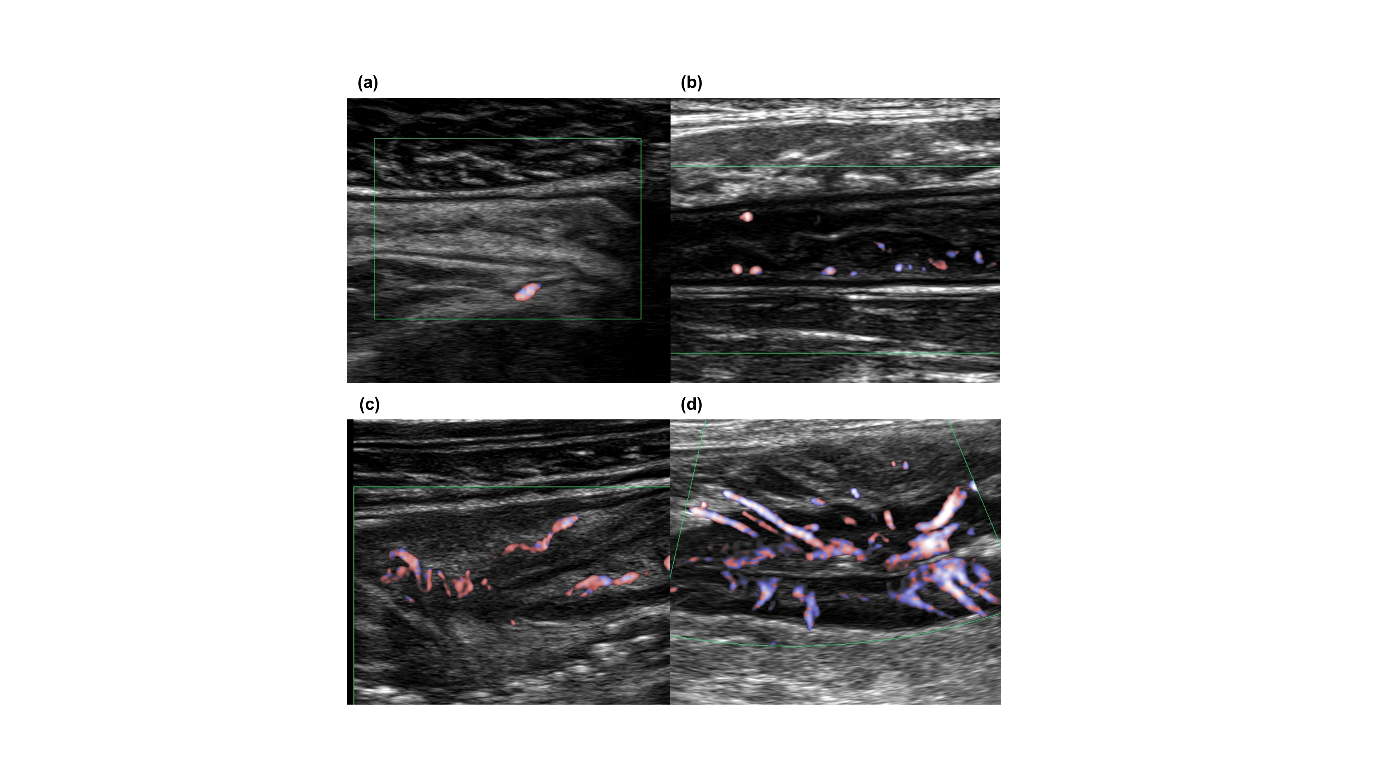


Figure S2.

Changes in the median HBI score and the proportion of patients achieving symptomatic remission. (A) Changes in the median HBI score in 18 patients with a baseline HBI score of ≥5. P-values were determined using the Wilcoxon signed-rank test (change from baseline, ^**^P < .01). Statistical multiplicity was adjusted using Bonferroni correction. (B) Proportion of patients achieving symptomatic remission (HBI <5) in the 18 patients with a baseline HBI score of ≥5. Missing values were imputed using the LOCF method. HBI = Harvey–Bradshaw index; CI = confidence interval; LOCF = Last-Observation-Carried-Forward.


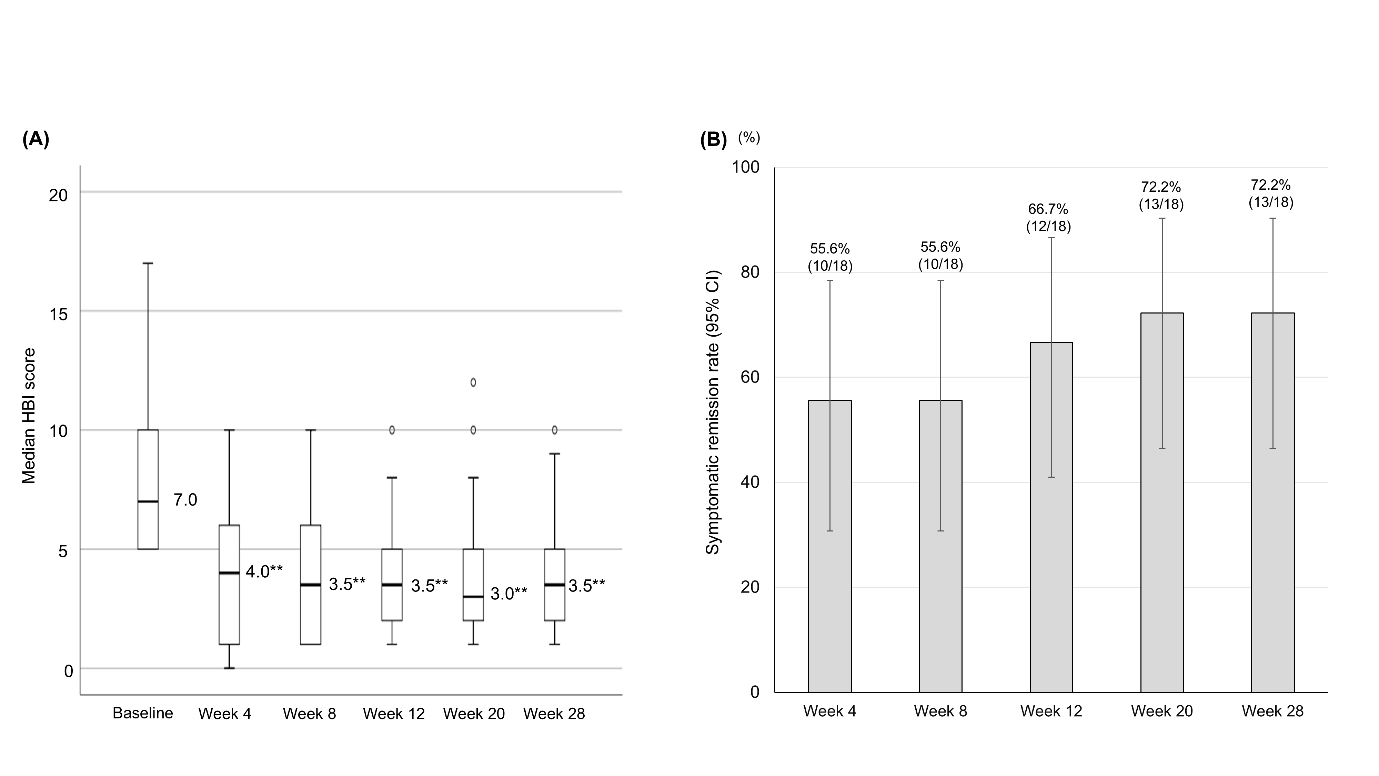


Figure S3.

Changes in the median C-reactive protein level and the median leucine-rich alpha-2 glycoprotein level. (A) Changes in the median C-reactive protein level. (B) Changes in the median leucine-rich alpha-2 glycoprotein level. Missing values were imputed using the LOCF method. P-values were determined using the Wilcoxon signed-rank test (change from baseline, ^**^P < .01, ^***^P < .001). Statistical multiplicity was adjusted using Bonferroni correction; LOCF = Last-Observation-Carried-Forward.


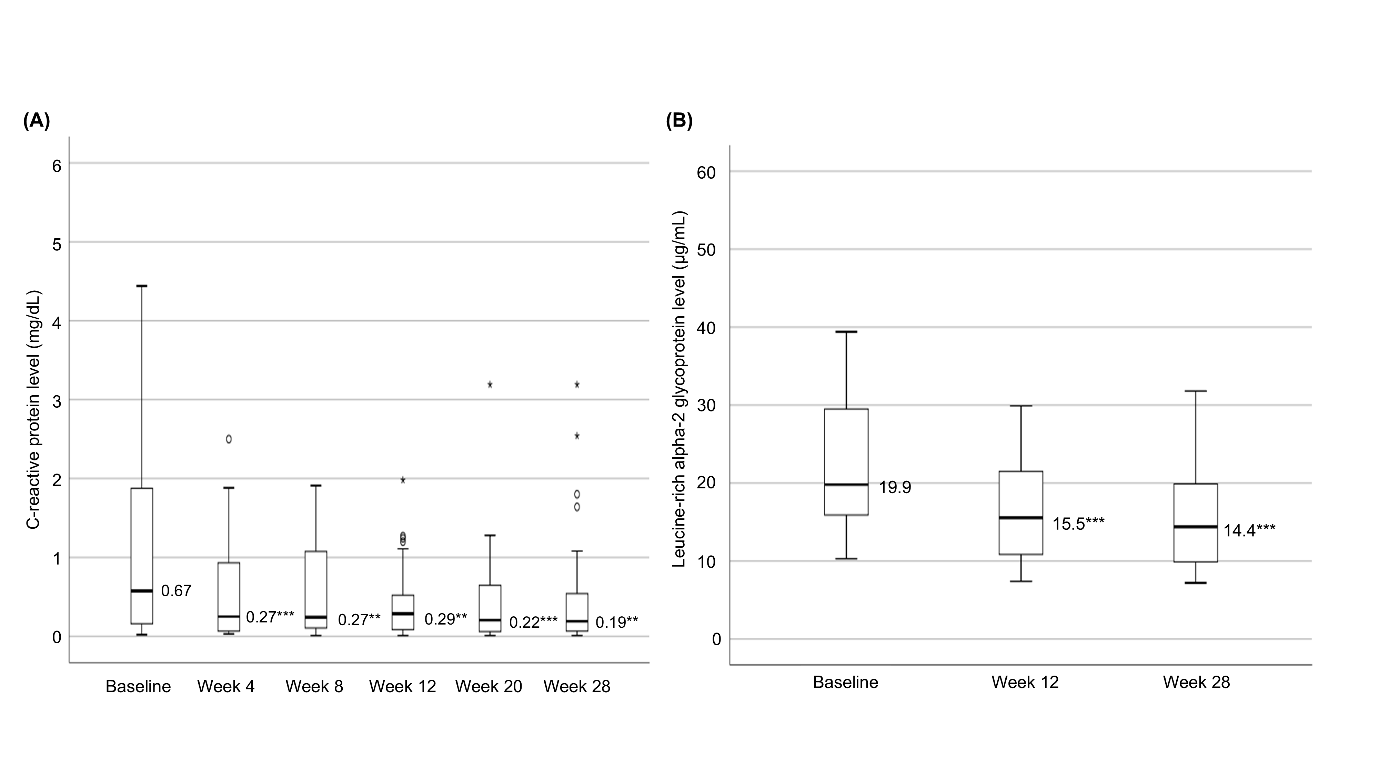


Figure S4.

Proportion of patients in whom drainage resolved and with active draining perianal fistulas. (A) Changes in the proportion of patients with resolved drainage among the 13 patients with active draining perianal fistulas at baseline. (B) Changes in the proportion of patients with active draining perianal fistulas in all 33 patients. Missing values were imputed using the LOCF method. CI = confidence interval; LOCF = Last-Observation-Carried-Forward.


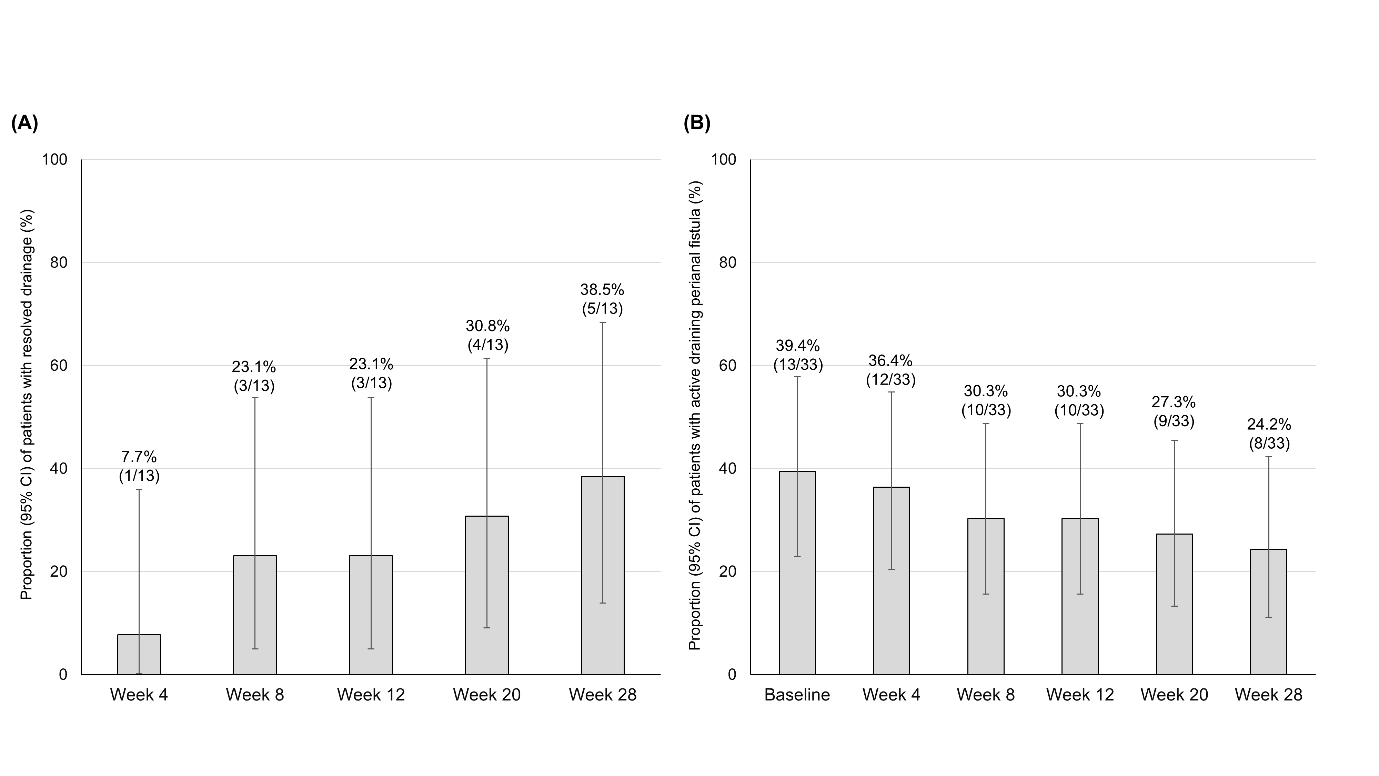


Figure S5.

Proportion of BWS and SMI grade stratified by biologic therapy history. (A) Changes in the proportion of patients with preserved BWS stratified by biologic therapy history. (B) Changes in the proportion of patients with SMI grades 0 and ≤1 stratified by biologic therapy history. Missing values were imputed using the LOCF method. P-values were determined using McNemar’s test (change from baseline, ^*^P < .05, ^**^P < .01). Statistical multiplicity was adjusted using Bonferroni correction. BWS = bowel wall stratification; SMI = superb microvascular imaging; CI = confidence interval; LOCF = Last-Observation-Carried-Forward.


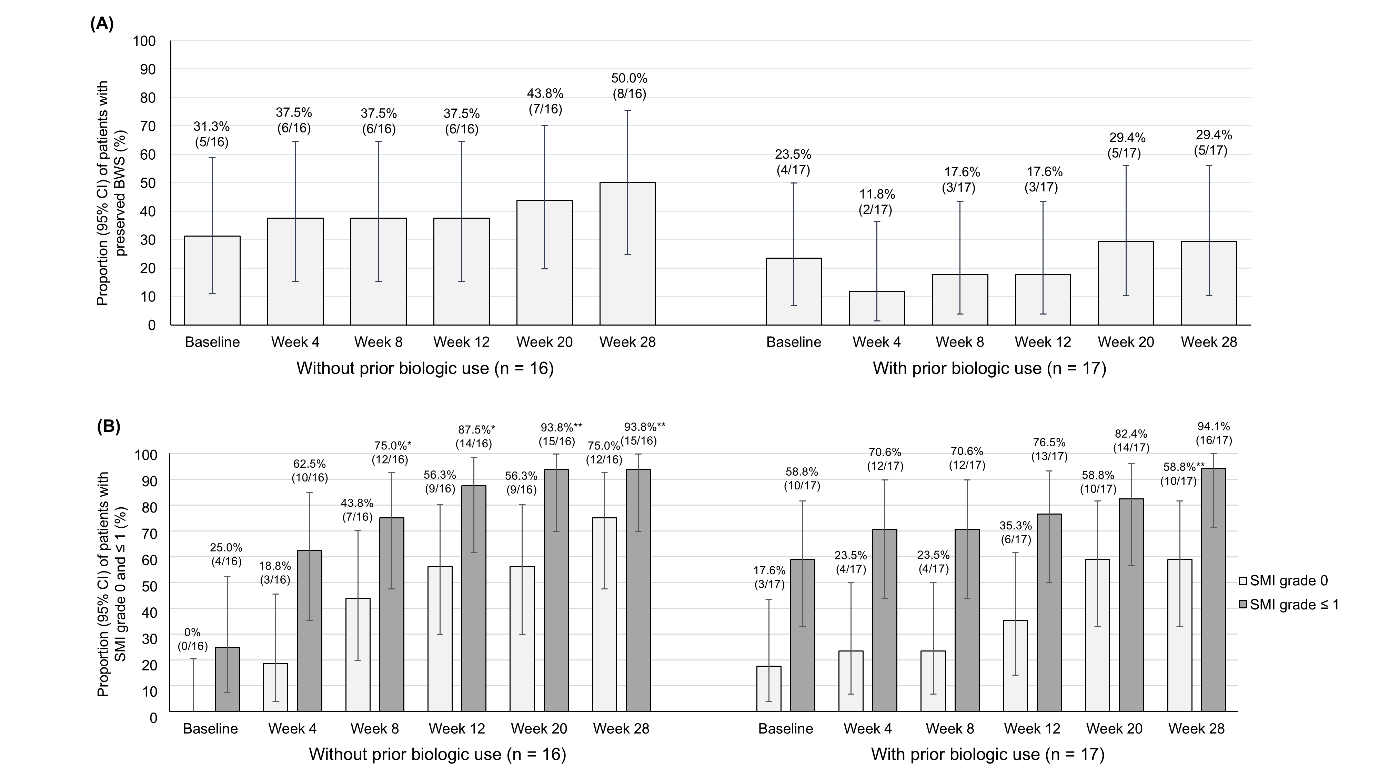


Figure S6.

Proportion of BWS and SMI grade stratified by the location of the representative lesion. (A) Changes in the proportion of patients with preserved BWS stratified by the location of the representative lesion at baseline. (B) Changes in the proportion of patients with SMI grades 0 and ≤1 stratified by the location of the representative lesion at baseline. Missing values were imputed using the LOCF method. P-values were determined using McNemar’s test (change from baseline, ^*^P < .05, ^**^P < .01). Statistical multiplicity was adjusted using Bonferroni correction. BWS = bowel wall stratification; SMI = superb microvascular imaging; CI = confidence interval; LOCF = Last-Observation-Carried-Forward.


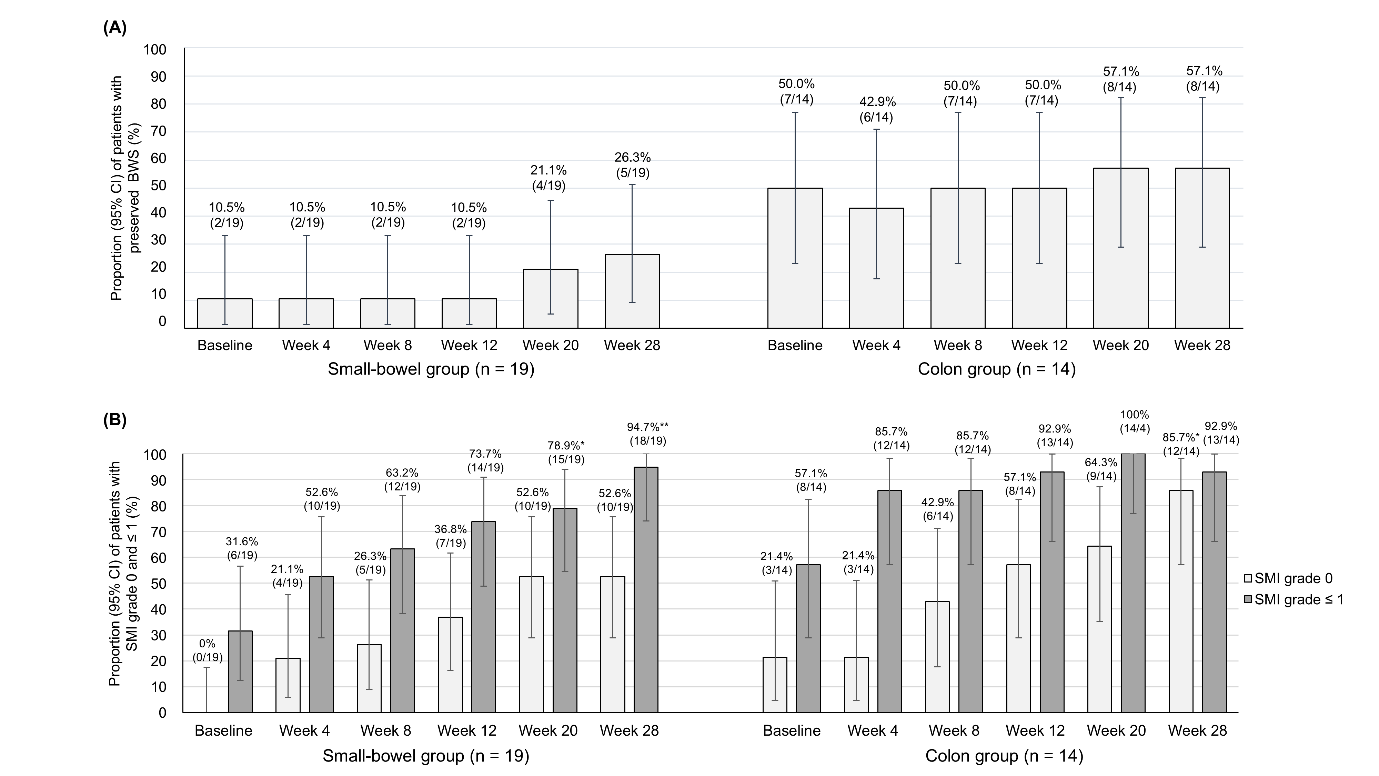


**Table S1.** Baseline characteristics of 33 patients with Crohn’s disease according to the previous medication of biologics.

|  | With prior biologic use  (n = 17) | Without prior biologic use  (n = 16) | *P-* value |
| --- | --- | --- | --- |
| Female / Male, n (%) | 5 (29.4%) / 12 (70.6%) | 3 (18.8%) / 13 (81.3%) | 0.688 |
| Median age, years (IQR) | 27.0 (24–37.5) | 26.0 (19.3–35.5) | 0.402 |
| Median disease duration, years (IQR) | 6.0 (3.0–10.5) | 0.0 (0.0–1.0) | <0.001 |
| Location of disease, n (%) |  |  |  |
| Ileitis | 1 (5.9%) | 1 (6.3%) | 0.367 |
| Ileocolitis | 14 (82.4%) | 15 (93.8%) |  |
| Colitis | 2 (11.8%) | 0 (0.0%) |  |
| Disease behaviour, n (%) |  |  |  |
| Non-stricturing, non-penetrating (B1) | 10 (58.8%) | 14 (87.5%) | 0.118 |
| Stricturing (B2) | 7 (41.2%) | 2 (12.5%) |  |
| Penetrating (B3) | 0 (0%) | 0 (0%) |  |
| Perianal disease, n (%) | 13 (76.5%) | 15 (93.8%) | 0.335 |
| Active perianal disease | 7 (41.2%) | 6 (37.5%) | 0.705 |
| Inactive perianal disease | 6 (35.3%) | 9 (56.3%) |  |
| Harvey–Bradshaw Index, median (IQR) | 4.0 (2.5–7.5) | 5.0 (4.0–7.8) | 0.309 |
| C-reactive protein, (mg/dl), median (IQR) | 1.30 (0.27–2.94) | 0.34 (0.14–2.18) | 0.292 |
| Albumin, (g/dl), median (IQR) | 3.8 (3.7–4.1) | 3.9 (3.7–4.3) | 0.444 |
| Leucine-rich alpha-2 glycoprotein, (μg/dl), median (IQR) | 21.2 (17.7–33.7) | 18.5 (15.6–27.3) | 0.217 |
| Most affected segment, n (%) |  |  |  |
| Small bowel | 10 (58.8%) | 9 (56.3%) | 1.000 |
| Colon | 7 (41.2%) | 7 (43.8%) |  |
| Bowel wall thickness (mm), median (IQR) | 5.5 (5.0–6.7) | 4.5 (4.2–5.2) | 0.006 |
| Bowel wall stratification, n (%) |  |  |  |
| Preserved | 4 (23.5%) | 5 (31.3%) | 0.708 |
| Loss | 13 (76.5%) | 11 (68.8%) |  |
| Superb microvascular imaging grade, n (%) |  |  |  |
| Grade 0 | 3 (17.6%) | 0 (0%) | 0.087 |
| Grade 1 | 7 (41.2%) | 4 (25.0%) |  |
| Grade 2 | 4 (23.5%) | 10 (62.5%) |  |
| Grade 3 | 3 (17.6%) | 2 (12.5%) |  |
| Concomitant medications, n (%) |  |  |  |
| Oral 5-aminosalicylic acid | 14 (82.4%) | 13 (81.3%) | 1.000 |
| Prednisolone | 5 (29.4%) | 10 (62.5%) | 0.084 |
| Budesonide | 5 (29.4%) | 2 (12.5%) | 0.398 |
| Immunomodulators | 9 (52.9%) | 1 (6.3%) | 0.007 |
| Elemental diet therapy | 6 (35.3%) | 7 (43.8%) | 0.728 |

IQR = interquartile range

**Table S2. Baseline characteristics of patients in the small bowel and colon groups.**

|  | Small bowel  (n = 19) | Colon  (n = 14) | *P-* value |
| --- | --- | --- | --- |
| Female / Male, n (%) | 5 (26.3%) / 14 (73.7%) | 3 (21.4%) / 11 (75.8%) | 1.000 |
| Median age, years (IQR) | 26.0 (21.0–36.0) | 27.0 (24.5–37.0) | 0.397 |
| Median disease duration, years (IQR) | 2.0 (0–7.0) | 3.5 (0.8–6.8) | 0.760 |
| Location of disease, n (%) |  |  |  |
| Ileitis | 2 (10.5%) | 0 (0%) | 0.122 |
| Ileocolitis | 17 (89.5%) | 12 (85.7%) |  |
| Colitis | 0 (0%) | 2 (14.3%) |  |
| Disease behaviour, n (%) |  |  |  |
| Non-stricturing, non-penetrating (B1) | 10 (52.6%) | 14 (100%) | 0.004 |
| Stricturing (B2) | 9 (47.4%) | 0 (0%) |  |
| Penetrating (B3) | 0 (0%) | 0 (0%) |  |
| Perianal disease, n (%) | 16 (84.2%) | 12 (85.7%) | 1.000 |
| Active perianal disease | 9 (47.4%) | 4 (28.6%) | 0.276 |
| Inactive perianal disease | 7 (36.8%) | 8 (57.1%) |  |
| Harvey–Bradshaw Index, median (IQR) | 3 (2–6) | 6 (4.8–8.5) | 0.011 |
| C-reactive protein, (mg/dl), median (IQR) | 0.34 (0.16–1.66) | 1.4 (0.3–4.1) | 0.132 |
| Albumin, (g/dl), median (IQR) | 3.9 (3.6–4.3) | 13.8 (3.7–4.0) | 0.287 |
| Leucine-rich alpha-2 glycoprotein, (μg/dl), median (IQR) | 18.9 (16.9–21.9) | 24.9 (15.4–33.9) | 0.358 |
| Bowel wall thickness (mm), median (IQR) | 5.2 (4.3–6.4) | 5.1 (4.4–5.9) | 0.872 |
| Bowel wall stratification, n (%) |  |  |  |
| Preserved | 2 (10.5%) | 7 (50.0%) | 0.019 |
| Loss | 17 (89.5%) | 7 (50.0%) |  |
| Superb microvascular imaging grade, n (%) |  |  |  |
| Grade 0 | 0 (0%) | 3 (21.4%) | 0.145 |
| Grade 1 | 6 (31.6%) | 5 (35.7%) |  |
| Grade 2 | 9 (47.4%) | 5 (35.7%) |  |
| Grade 3 | 4 (21.1%) | 1 (7.1%) |  |
| Concomitant medications, n (%) |  |  |  |
| Oral 5-aminosalicylic acid | 16 (84.2%) | 11 (78.6%) | 0.678 |
| Prednisolone | 8 (42.1%) | 7 (50.0%) | 0.733 |
| Budesonide | 5 (26.3%%) | 2 (14.3%) | 0.670 |
| Immunomodulators | 8 (42.1%) | 2 (14.3%) | 0.131 |
| Azathioprine | 7 (36.8%) | 1 (7.1%) | 0.098 |
| 6-mercaptopurine | 1 (5.3%) | 1 (7.1%) | 1.000 |
| Elemental diet therapy | 6 (31.6%) | 7 (50.0%) | 0.472 |
| Previous medications, n (%) |  |  |  |
| Infliximab | 4 (21.1%) | 3 (21.4%) | 1.000 |
| Adalimumab | 7 (36.8%) | 5 (35.7%) | 1.000 |
| Ustekinumab | 3 (15.8%) | 4 (28.6%) | 0.422 |
| Vedolizumab | 1 (5.3%) | 3 (21.4%) | 0.288 |
| Biologics | 10 (52.6%) | 7 (50.0%) | 1.000 |

IQR = interquartile range

**Table S3.** Adverse events occurring during the study period

| **Adverse events, n (%)** | |
| --- | --- |
| Headache | 1 (3.0) |
| Nausea | 1 (3.0) |
| Phlebitis | 1 (3.0) |
| Bowel obstruction | 2 (6.1) |
| Exacervation of Crohn’s disease | 2 (6.1) |
| **Adverse events leading to the discontinuation of Risankizumab, n (%)** | |
| Exacervation of Crohn’s disease | 2 (6.1) |
